# Gold/DNA-Cu^2+^ Complex Nanozyme-Based Aptamer Lateral Flow Assay for Highly Sensitive Detection of Kanamycin

**DOI:** 10.3390/molecules29194569

**Published:** 2024-09-26

**Authors:** Xiuping Li, Rui Chang, Shengmei Tai, Minxin Mao, Chifang Peng

**Affiliations:** 1 State Key Laboratory of Food Science and Resources, Jiangnan University, Wuxi 214122, China; 6200112045@stu.jaingnan.edu.cn (X.L.); 6180111003@stu.jiangnan.edu.cn (R.C.); 7220112046@stu.jiangnan.edu.cn (S.T.); 2 School of Food Science and Technology, Jiangnan University, Wuxi 214122, China; 3 Shandong Institute of Pomology, Taian 271000, China; 15161521628@163.com; 4 International Joint Laboratory on Food Safety, Jiangnan University, Wuxi 214122, China

**Keywords:** aptamer, lateral flow assay, nanozyme, kanamycin

## Abstract

Aptamer-based lateral flow analysis (Apt-LFAs) has promising applications in many fields. Nanozymes have demonstrated high potential in improving the performance of Apt-LFAs and have been increasingly utilized in recent studies. In this study, we developed a nanozyme-based Apt-LFA for the rapid and sensitive detection of kanamycin by using a novel dual-functionalized AuNPs@polyA-DNA/GpG-Cu^2+^ nanozyme as a nanoprobe. In the nanoprobe design, the polyA-cDNA strand can discriminate a kanamycin aptamer from the kanamycin/aptamer complex, and the GpG-Cu^2+^ complex can amplify the detection signal by catalyzing the chromogenic reaction. The nanozyme Apt-LFA can quantify kanamycin in the range of 1–250 ng/mL with an LOD of 0.08 ng/mL, which demonstrated a 4-fold sensitivity improvement and had a wider linear range than the conventional AuNP-based LFA. The Apt-LFA was successfully applied to the detection of kanamycin in honey with good recoveries. Our dual-functionalized AuNP nanoprobe is easily prepared and can be highly compatible with the conventional AuNP-DNA-based LFA platform; thus, it can be extended to the application of Apt-LFAs for other small molecules.

## 1. Introduction

In recent years, lateral flow assays (LFAs) have attracted a great deal of interest owing to their advantages of fast turnaround time, simplicity of operation, and low cost [1,2]. However, to date, these LFAs have mainly used antibodies as target recognition elements [3]. The rapid development of aptamer technology offers the possibility of circumventing the disadvantages of poor pH and thermal stability, high cost, and batch variation in antibody-based LFAs [4]. Aptamers are single-stranded oligonucleotides that offer advantages such as thermal stability, low cost, and ease of modification as recognition elements [2,5]. Therefore, aptamer-based LFAs (Apt-LFAs) have attracted much interest from researchers and have been developed to detect a variety of targets [6]. Additionally, we recently reported the design of an AuNPs@polyA-cDNA nanoprobe for the rapid detection of acetamiprid [7].

In LFAs, gold nanoparticles (AuNPs) are widely used as signal reporters [8,9,10]. However, the low sensitivity of many LFAs has greatly limited their application in the detection of ultratrace analytes [11,12]. In recent years, numerous methods have been developed to improve the sensitivity of LFAs [8]. Due to their good stability and cost-effectiveness, nanozymes, which can catalyze the oxidation of substrates and produce intense color enhancement [13,14], have received considerable attention in the development of LFAs [15,16]. For example, Tatsuya et al. developed an LFA using colloidal palladium nanoparticles (PdNPs) as the signal reporters, with 3,3′,5,5′-tetramethylbenzidine (TMB) and 3,3′-diaminobenzidine (DAB) as oxidation substrates to produce signal amplification [17]. Han et al. developed a highly sensitive LFA for the detection of Escherichia coli O157: H7 by using palladium–platinum (Pd-Pt) nanoparticles as nanozyme probes and TMB as an oxidation substrate [18]. Liu et al. developed a magnetic Prussian blue nanozyme (MPBN)-mediated dual-readout lateral flow immunoassay with TMB as an oxidation substrate for the highly sensitive detection of ractopamine (RAC) and clenbuterol (CLE) [19]. Cheng et al. developed a dual lateral flow immunoassay (LFIA) based on mesoporous core–shell palladium@platinum (Pd@Pt) nanoparticles for the highly sensitive detection of *Salmonella enteritidis* and *Escherichia coli* O157:H7 [20]. Zhao et al. developed a sandwich lateral flow immunoassay using PtPd nanoparticles as colorimetric probes for the highly sensitive detection of enzymatic inhibition and phosphorylation [21]. Although many nanozymes have been utilized to improve the performance of antibody-based LFAs, very few nanozyme-based Apt-LFAs have been reported [14,22]. In this Apt-LFA for CA125, CA125 glycoprotein-labeled AuNPs were directly used as nanozymes [23]. However, the AuNPs commonly demonstrated very weak peroxidase-like activity, making it difficult to achieve significant signal amplification with this strategy [12,24].

We hypothesized that one of the challenges in developing an effective nanozyme-based Apt-LFA is to reduce the surface masking effect in DNA-modified noble metal nanozymes, which significantly impairs catalytic activity. Kim et al. used the correlation between catalytic properties and the exposed surface area of nanoparticles to achieve the highly sensitive colorimetric detection of lysozymes [25]. Weerathunge et al. utilized acetamiprid aptamers to shield the surface of GNPs to inhibit nanozyme activity, and in the presence of acetamiprid, these aptamers left the surface of the GNPs in a target concentration-dependent manner, reactivating GNP nanozyme activity [26]. In addition, Weerathunge et al. achieved the first detection of the infective murine norovirus (MNV) equivalent to the lower end of the ID50 for NoV within a few minutes after replacing the acetamiprid aptamer with the MNV aptamer [27]. Furthermore, Sharma et al. proposed an ultra-fast and highly sensitive biosensing method for the detection of kanamycin by utilizing kanamycin to compete with adsorbed aptamers on the surface of GNPs, thereby “turning on” the nanozyme activity of GNPs [28]. It was reported that coper ions (Cu^2+^) can catalyze a Fenton reaction in which highly reactive hydroxyl radicals (·OH) are produced [29]. Based on this catalytic activity of Cu^2+^, Chang et al. developed the detection of glyphosate in water [30]. In addition, Li et al. constructed a DNA-based metalloenzyme through a unique coordination pattern between Cu^2+^ and a G-rich DNA duplex (duplex-GpG-Cu^2+^). The DNA scaffold was found to greatly accelerate the Cu(II)-catalyzed oxidation reaction, enabling the highly sensitive detection of Cu^2+^ in water with an LOD of 1.2 nM [31].

Inspired by the properties of the DNA/Cu^2+^ complex in peroxidase mimetics, we prepared a dual-functionalized AuNPs@polyA-DNA/GpG-Cu^2+^ nanoprobe, in which the polyA-DNA strand can discriminate between a kanamycin aptamer and the kanamycin/aptamer complex, while the GpG-Cu^2+^ complex can simultaneously amplify the detection signal. This design may effectively circumvent the surface masking effect common in nanozymes. Kanamycin is a widely used aminoglycoside bactericidal antibiotic, which is strictly regulated for use as a veterinary drug and feed additive [32,33]. The LFA for kanamycin is very valuable for monitoring drug residues in food [34,35]. Based on this novel nanoprobe design, highly sensitive detection of kanamycin was achieved. Additionally, the Apt-LFA can be easily adapted for the detection of other small molecules simply by changing the aptamer and polyA-DNA strands.

## 2. Results

### 2.1. Principle of the Apt-LFA

**Scheme 1:**
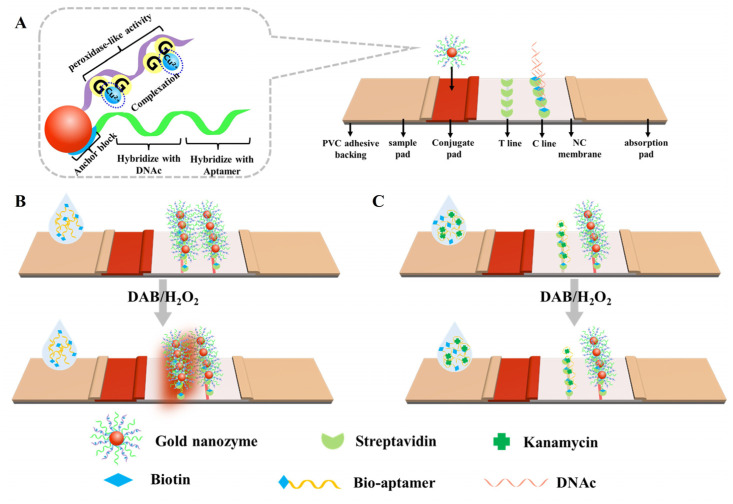
Schematic representation of kanamycin detection via Apt-LFA. (**A**) Structure of gold nanozyme and LFA strip; (**B**) negative result of Apt-LFA detection (before and after DAB/H_2_O_2_ treatment); (**C**) positive result of Apt-LFA detection (before and after DAB/H_2_O_2_ treatment).

The structure of the signal probe is shown in Scheme 1A. The signal probe is modified with two DNA strands: polyA-DNA and SH-DNA. The polyA-DNA has three functional regions. The first is a polyA segment consisting of 15 adenine bases, which are anchored to the AuNPs by a non-covalent interaction. The second region is complementary to the DNAc strand located on the C line of the strip. The third region is partially complementary to the aptamer strand. SH-DNA consists of an A(G4C)3G5C base sequence, which has a unique complexation with Cu^2+^ and facilitates the binding of Cu^2+^ to the signal probe, thereby enhancing peroxidase activity.

This test strip design leverages the fact that when the nucleic acid aptamer strand binds to kanamycin, it reduces or prevents the aptamer from binding to the polyA-DNA on the signal probe. Additionally, the signal amplification of the strip is based on the signal probe, which catalyzes DAB in the presence of H_2_O_2_, producing a sepia-colored product. As shown in Scheme 1B, when a negative sample solution (without kanamycin) is applied, the biotinylated aptamer binds to the polyA-DNA on the signal probe to form conjugates. These conjugates are captured by streptavidin (SA) on the T line, enriching the signal probes at the T line and producing a clear sepia band when catalyzed by the signal probes after H_2_O_2_ and DAB are added dropwise. As shown in Scheme 1C, when a positive sample solution (containing kanamycin) is applied, kanamycin binds to the aptamer, making it difficult for SA on the T line to capture the signal probe, thus reducing the ability to catalyze DAB and resulting in a lighter or not visible T line. In addition, the polyA-DNA of the signal probe can be captured by DNAc on the C line, regardless of whether kanamycin is present in the sample solution.

### 2.2. Optimization of the Apt-LFA

A series of parameters for the Apt-LFA were systematically optimized to achieve optimal conditions. These parameters included the concentrations of H_2_O_2_, DAB, copper acetate, and aptamer; the ratio of polyG to polyA-DNA; the number of washes for preparing gold nanomimetic enzyme signal probes; and the reaction time of the test strips. The relative T/C signal intensity, measured by a colloidal gold test strip quantitative analyzer, was used as an optimization indicator.

#### 2.2.1. Optimization of H_2_O_2_ and DAB Concentration

The concentrations of H_2_O_2_ and DAB affect the signal intensity of the T line, so they were optimized to achieve the best performance of the Apt-LFA. As shown in Figure S2A, the relative signal intensity (T/C) increased as the concentration of H_2_O_2_ increased, reaching its maximum at 20%. As shown in Figure S2B, a DAB concentration of 5 mg/mL produced clear T and C lines. Therefore, 20% H_2_O_2_ and 5 mg/mL DAB were selected for this Apt-LFA.

#### 2.2.2. Optimization of Cu^2+^ Concentration

**Figure 1:**
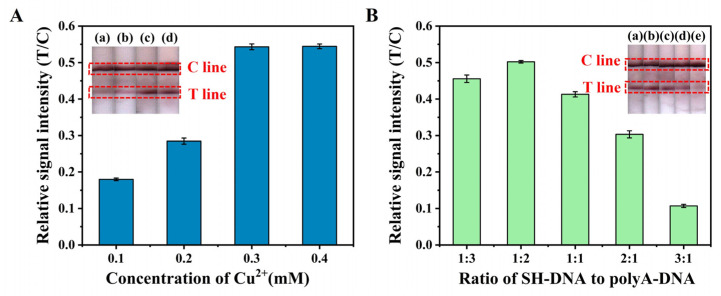
(**A**) Optimization of Cu^2+^ concentration. Inset: photographs of Apt-LFAs with different DAB concentrations. (a), (b), (c), and (d) are 0.1 mM, 0.2 mM, 0.3 mM, and 0.4 mM, respectively (*n* = 3). (**B**) Optimization of ratio of SH-DNA to polyA-DNA. Inset: photographs of Apt-LFAs with different ratios of SH-DNA to polyA-DNA. (a), (b), (c), (d), and (e) are 1:3, 1:2, 1:1, 2:1, and 3:1, respectively (*n* = 3).

The concentration of Cu^2+^ affects the catalytic capacity of the nanozymes for DAB, which, in turn, influences the signal intensity of the T line. To determine the optimal copper acetate concentration, different concentrations (0.1 mmol/L, 0.2 mmol/L, 0.3 mmol/L, and 0.4 mmol/L) were tested in optimization experiments. As shown in Figure 1A, the relative signal intensity (T/C) gradually increased with the concentration of Cu^2+^. The highest T/C signal was achieved at a Cu^2+^ concentration of 0.3 mmol/L.

#### 2.2.3. Optimization of the SH-DNA-to-polyA-DNA Ratio

The ratio of SH-DNA to polyA-DNA affects not only the ability to bind the aptamer but also the amount of Cu^2+^ loading, making it an important parameter of the Apt-LFA. As shown in Figure 1B, the effect of different SH-DNA to polyA-DNA ratios (1:3, 1:2, 1:1, 2:1, 3:1) on T line signal intensity was investigated. The results showed that the highest T/C signal was obtained when the ratio of SH-DNA to polyA-DNA was 1:2.

#### 2.2.4. Optimization of Aptamer Concentration

**Figure 2:**
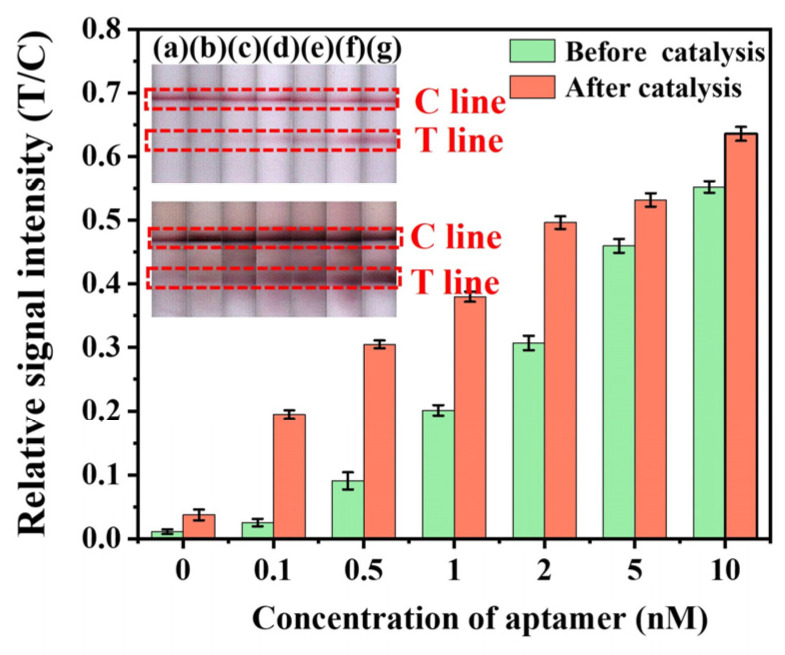
Optimization of aptamer concentration. Inset: photographs of Apt-LFA before and after catalysis with different aptamer concentrations. (a), (b), (c), (d), (e), (f), and (g) are 0 nM, 0.1 nM, 0.5 nM, 1 nM, 2 nM, 5 nM, and 10 nM, respectively (*n* = 3).

Aptamer concentration can greatly influence the sensitivity of the Apt-LFA. To determine the optimal aptamer concentration, test strips were treated with various aptamer concentrations (0 nM, 0.1 nM, 0.5 nM, 1 nM, 2 nM, 5 nM, and 10 nM) while maintaining the concentration of AuNPs@polyA-cDNA/GpG-Cu^2+^ nanozymes at 0.5 nM. As shown in Figure 2, a strong T/C signal was obtained with 10 nmol/L aptamer when the test was carried out without catalytic substrate color enhancement. After signal amplification, a similarly strong T/C signal was achieved with 2 nmol/L aptamer. Therefore, 2 nmol/L aptamer was selected for this Apt-LFA.

### 2.3. Performance of Apt-LFA

#### 2.3.1. Detection of Kanamycin

**Figure 3:**
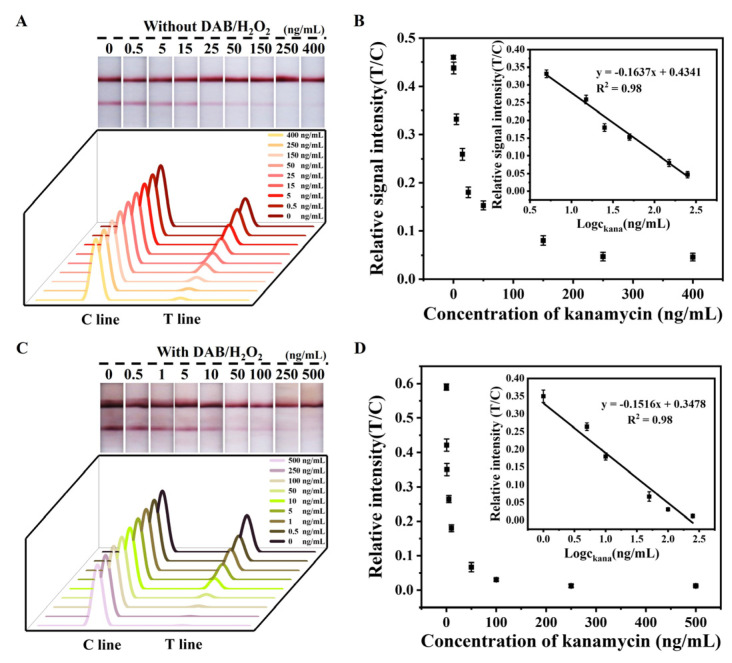
Results obtained under uncatalyzed conditions: (**A**) signals obtained from strip reader and photographs of Apt-LFA without substrate-catalyzed amplification; (**B**) plots of T/C signal versus kanamycin concentration (*n* = 3). Results obtained under catalyzed conditions (with DAB/H_2_O_2_): (**C**) signals obtained from strip reader and photographs of Apt-LFA with substrate-catalyzed amplification; (**D**) plots of T/C signal versus the kanamycin concentration (*n* = 3).

As shown in Figure 3A,B, in the conventional Apt-LFA without the use of a catalytic substrate for color enhancement, the logarithm of the T/C signal versus kanamycin concentration exhibits a linear relationship in the range of 5–250 ng/mL. Based on 3σ/k (σ is the standard deviation of the blank control and k is the slope of the linear equation), the LOD was calculated to be 0.3 ng/mL.

After catalytic substrate color enhancement, the color development in the T lines was deepened to some extent. Additionally, the logarithm of the T/C signal versus kanamycin concentration exhibits a strong linear relationship in the range of 1–250 ng/mL (Figure 3C,D). The LOD was calculated to be 0.08 ng/mL. Therefore, the sensitivity of the Apt-LFA was improved fourfold, and the linear range was also significantly widened. The sensitivity of our proposed method is comparable to, or even higher than, other colorimetric LFAs with complex sensing strategies and homogeneous colorimetric/fluorescent methods, with the exception of a homogeneous fluorescent method involving DNA amplification (Table S2).

#### 2.3.2. Specificity Analysis

**Figure 4:**
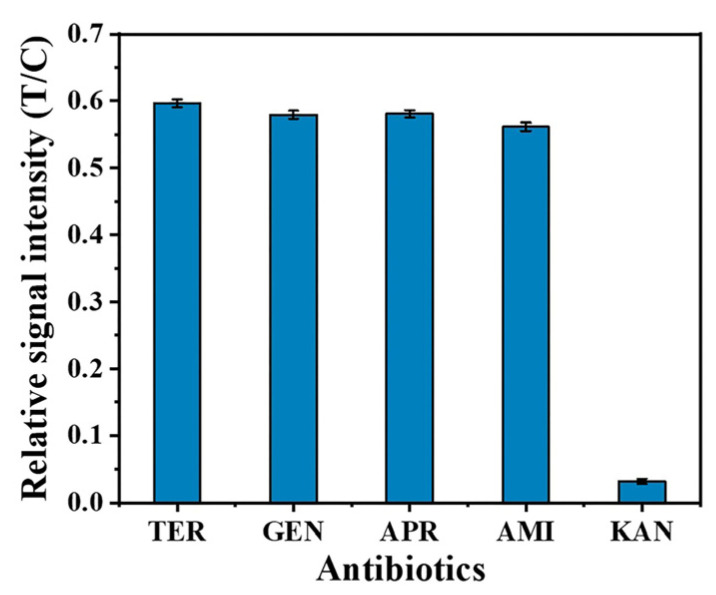
Selectivity of the assay for different antibiotics (TER, GEN, APR, AMI, and KAN) (*n* = 3).

To assess the selectivity of the Apt-LFA for kanamycin, various other antibiotics, including hygromycin, gentamicin sulfate, acamycin, apomycin, and kanamycin, were tested using the Apt-LFA to verify its specificity. As shown in Figure 4, all antibiotics were tested at a concentration of 100 ng/mL, and only kanamycin showed significant signal inhibition in the T line region. These results confirm the high specificity of the Apt-LFA.

#### 2.3.3. Real Sample Assay

To verify the practical performance of the Apt-LFA, the test strips were used to analyze honey samples spiked with different concentrations of kanamycin (Table 1). The recoveries ranged from 95.3% to 105.5%, with experimental relative standard deviations (RSDs) between 5.5% and 8.1%. The method meets the EU requirement of 150 μg/kg for the maximum residue limits (MRLs) of kanamycin in milk. Therefore, the Apt-LFA enables the detection of kanamycin in real samples with high accuracy and reliability.

## 3. Materials and Methods

### 3.1. Chemicals and Apparatus

The details of the chemicals and apparatus utilized are described in the Supplementary Materials.

### 3.2. Preparation of DNA-AuNPs Conjugates and AuNPs Nanozyme

Citrate-reduced AuNPs with a diameter of 15 nm [36,37] were utilized in the preparation. The AuNPs@polyA-DNA nanoprobe was prepared using a slightly modified “low pH” method [38]. The AuNPs@polyA-cDNA/GpG-Cu^2+^ nanozyme was similarly obtained, except for an additional step of loading Cu^2+^ ions through complexation with the GpG strand. The details are described in the Supplementary Materials.

### 3.3. Fabrication of the Apt-LFA Test Strip

The Apt-LFA test strip consisted of four parts, the sample pad, conjugate pad, nitrocellulose membrane (NC membrane), and absorbent pad, which were overlapped and attached to a PVC adhesive backing. The total length of the test strip was 60 mm and the width was 4 mm. The sample pad had a length of 18 mm, the conjugate pad had a length of 8 mm, the NC membrane had a length of 25 mm, and the absorbent pad had a length of 15 mm. The overlap between the parts was 2 mm. The AuNPs@polyA-cDNA/GpG-Cu^2+^ nanozymes (25 nM, 1.4 μL) were spread on the conjugate pad (Scheme 1A). Streptavidin (SA) solution and streptavidin-biotinylated DNA strands were placed on the T and C lines of the NC membrane, respectively. The distance between the T and C lines was 5 mm. Finally, the Apt-LFA strips were stored at 4 °C.

### 3.4. Protocol of the Apt-LFA for Kanamycin

The aptamer solution was mixed with different concentrations of kanamycin and incubated at 25 °C for 20 min. Running buffer (4 × SSC, 0.5% Tween 20, pH 7) was added to the incubated solution. The mixture (70 μL) was then added to the sample pad, and after 5 min, 2 μL of the DAB and H_2_O_2_ mixture was added dropwise to the T line of the test strip. The reaction was allowed to proceed for 3 min before scanning and recording the experimental results. Qualitative and quantitative analyses were performed using visual inspection and a colloidal gold test strip quantitative analyzer.

### 3.5. Sample Meaurements

Honey samples were diluted by a factor of 10 and filtered through a 0.22 µm microporous membrane. Then, different concentrations of kanamycin (5 ng/mL, 50 ng/mL, and 100 ng/mL) were added. An amount of 1 µL of aptamer (0.2 μmol/L) was mixed with 99 µL of the pretreated honey solution, incubated for 20 min, and then applied to the above test strips to detect kanamycin. Five minutes later, 2 µL of the DAB and H_2_O_2_ mixture was added dropwise to the T line. After an additional 3 min, quantitative analysis was carried out using a colloidal gold strip quantitative analyzer.

## 4. Conclusions

An Apt-LFA based on a novel dual-functionalized AuNPs@polyA-cDNA/GpG-Cu^2+^ nanozyme was successfully developed for the rapid and sensitive detection of kanamycin. The GpG-Cu^2+^ complex in the nanoprobe amplifies the detection signal through a catalytic color reaction. The dual-functionalized design of the nanozyme is easy to achieve and has excellent compatibility with conventional DNA-AuNP LFAs. The nanozyme Apt-LFA is more sensitive and has a wider linear range than the conventional AuNP-based LFA. This nanozyme probe design can be easily extended to detect other small molecules in Apt-LFA simply by changing the polyA-DNA, making it highly promising for point-of-care testing (POCT) applications.

## Figures and Tables

**Table 1:** Recovery results of spiked kanamycin in honey samples (*n* = 3).

Spiked (ng mL^−1^)	Detected (ng mL^−1^)	Recovery (%)	RSD (%)
0	ND ^a^	-	-
50	48	95.3	5.5
500	528	105.5	8.1
1000	1046	104.6	6.0

^a^ Not detected.

## Data Availability

Data are contained within the article and Supplementary Materials.

## Supplementary Materials

The following supporting information can be downloaded at: https://www.mdpi.com/article/10.3390/molecules29194569/s1: Materials and Apparatus; Preparation of Gold Nanozymes; Preparation of SA-DNAC Conjugates. Figure S1: (A) TEM images of AuNPs. (B) UV–vis absorption spectra of AuNPs and AuNPs@polyA-DNA/SH-DNA. Figure S2: (A) Optimization of H_2_O_2_ concentration and (B) Optimization of DAB concentration. Inset: photographs of Apt-LFAs with different DAB concentrations. Table S1: Nucleic acid sequence for kanamycin Apt-LFA. Table S2: Comparison of analytical properties of various kanamycin sensors. Refs. [39,40,41,42] are cited in Supplementary Materials.
