# Supplementary material for: Gold/DNA-Cu2+ Complex Nanozyme-Based Aptamer Lateral Flow Assay for Highly Sensitive Detection of Kanamycin
Source: Molecules. 2024 Sep 26;29(19):4569. doi: 10.3390/molecules29194569 (PMC11478301; doi:10.3390/molecules29194569)
Supplement: Supplementary file 1 [file molecules-29-04569-s001.zip › molecules-3190154-supplementary.pdf]

Supporting Information

Gold/DNA-Cu^{2+} Complex Nanozyme-based Aptamer Lateral Flow Assay for Highly Sensitive Detection of Kanamycin

XiuPing Li [1,2], Rui Chang [1,2], Shengmei Tai [1,2] and Chifang Peng [1,2,3,*]

1 State Key Laboratory of Food Science and Resources, Jiangnan University, Wuxi, 214122, China
2 School of Food Science and Technology, Jiangnan University, Wuxi, 214122, China
3 International Joint Laboratory on Food Safety, Jiangnan University, Wuxi, 214122, China
* Correspondence: pcf@jiangnan.edu.cn; Tel.: +86-510-85329081

1. Materials and Apparatus

The 21-mer kanamycin-binding aptamer (5'- TGGGG GTTGA GGCTA AGCCG A -3') with the apparent dissociation constant (Kd) estimated to be 78.8 nM was adopted from the literature32. All oligonucleotides purified by HPLC were synthesized by Shanghai Sangon Biotechnology Co. Ltd. (Shanghai, China) (Table S1, Table S2).

Chloroauric acid (HAuCl4), trisodium citrate and copper acetate monohydrate were purchased from Sigma-Aldrich (St. Louis, MO, USA). 3,3'-Diaminobenzidine (DAB) was purchased from Beijing Enochai Co. Ltd. (Beijing, China). Hydrogen peroxide (H2O2), disodium hydrogen phosphate dodecahydrate, sodium dihydrogen phosphate dihydrate, trisodium phosphate, sucrose and Tween-20 were purchased from Sinopharm Chemical Reagent Co. Ltd. (Shanghai, China). Kanamycin, bovine serum albumin (BSA) and streptavidin (SA) were ordered from Sangon Biotech Co. Ltd. (Shanghai, China). All reagents were of analytical grade and used as received. All solutions were prepared with ultrapure water (18.2 MΩ/cm) from a Millipore Milli-Q water purification system (Billerica, MA).

The absorbent pad (SX27), sample pad (SB06), Conjugate pad (VL78), nitrocellulose (NC) membrane (Sartorius CN140), and PVC plastic adhesive backing used in the experiments were purchased from Shanghai Jieyi Biotechnology Co. Ltd (Shanghai, China). The ultrafiltration tubes are biosharp-0.5 mL 30 K ultrafiltration centrifuge tubes from LABGIC (Beijing, China). Each lateral flow assay strip was dispensed and cut using XYZ 3050 dispensing platform and KM-3100 Cutter (BioDot, Irvine, CA, USA). The signals of the test strip were scanned by a ZJ-600 multi-channel colloidal gold reader (Zhongde, Wuxi). Zetasizer Nano ZS (Malvern, UK) was used to determine hydro-diameter. A transmission electron microscope (TEM) image was obtained using an electron microscope of JEM-2100 (Japan Electronics Corporation, Japan) operated at 200 kV.

2.2 Preparation of gold nanozymes

The sodium citrate reduction method used for the preparation of AuNPs with an average diameter of 15 nm was prepared according to the reported method with slight modifications[1, 2]. 100 mL of 0.01% HAuCl4 was added to a 250 mL conical flask,

heated and stirred until the solution burst into a boil, and kept for 1 min-2 min. Subsequently, 2 mL (1%) of trisodium citrate solution was quickly added, heated and stirred were continued. The color of the solution gradually changed from light yellow to dark purple and finally to burgundy, and kept heated for 10 min, cooled at room temperature and refrigerated at 4°C for backup. The glassware used for the synthesis and stocking of nanomaterials was soaked in aqua regia (hydrochloric acid: nitric acid = 3:1) for 12 h, washed with ultrapure water and then used.

We used the traditional "low pH" method to construct AuNPs@polyA-DNA/SH-DNA signal nanoprobe[3]. A small amount (i.e. 5-15 μL) of 100 μM nucleic acids (polyA-DNA mixed with SH-DNA in a certain ratio) were added to 1000 μL of prepared AuNP solution (10 nM) and mixed by brief vortex mixing, followed by the addition of 20 μL of citrate-HCl buffer (500 mM, pH 3). After a brief vortex mixing, the samples were allowed to incubate for 3 min at room temperature. The pH of the AuNP solution was then adjusted back to neutral by adding 60 μL of 500 mM HEPES buffer (pH 7.6). The sample was allowed to incubate for another 5−10min at room temperature. Finally, the AuNPs@polyA-DNA mixture was centrifuged at 10,000 r/min for 20 min and the supernatant was removed. The precipitate was resuspended in resuspension solution (20 mM Na_3PO_4, 5% BSA, 10% sucrose, 0.25% Tween-20), centrifuged at 10,000 r/min for 20 min, and centrifuged three times to completely remove the free cDNA strands. And finally, the AuNPs@polyA-DNA/SH-DNA was re-dispersed in 400 μL resuspension solution, the concentration of AuNPs@polyA-DNA/SH-DNA was 25 nM.

The prepared AuNPs@polyA-DNA/SH-DNA was mixed with 0.3 mM copper acetate at a 1:1 ratio and incubated for a specified time. The mixture was then centrifuged at 6000 rpm for 10 minutes to remove unbound copper acetate. Concurrently, the volume of the resulting AuNPs@polyA-cDNA/GpG-Cu^{2+} nanozymes was concentrated to half of the original mixture volume (the concentration of AuNPs@polyA-cDNA/GpG-Cu^{2+} was 25 nM) and subsequently stored at 4°C.

3. Preparation of SA-DNA_C conjugates

DNA_C was designed as a capture probe to capture gold nanozymes at the C line. To immobilize DNA_C at the C line, a streptavidin-DNA_C conjugate was prepared using biotinylated DNA and streptavidin via the high affinity between streptavidin and biotin. Specifically, 100 μL (2.5 mg/mL) of SA was incubated with 100 μL (250 μmol/L) of biotinylated DNA_C at 4℃ for 1 h to complete the binding of SA to Biotin-DNAC. To remove the free biotinylated nucleic acid, centrifugation was performed for 20 min at 6000 r/min using an ultrafiltration tube (MWCO 30 kDa), centrifuged three times, and resuspended in 300 μL (10 mM) PBS. The prepared conjugates were stored at 4°C.

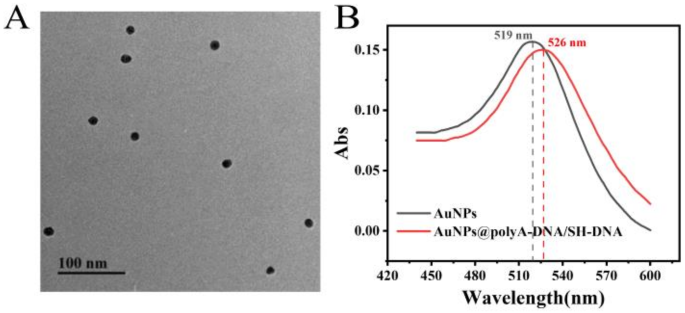

Figure S1. (A) TEM images of AuNPs. (B) UV–vis absorption spectra of AuNPs and AuNPs@polyA-DNA/SH-DNA.

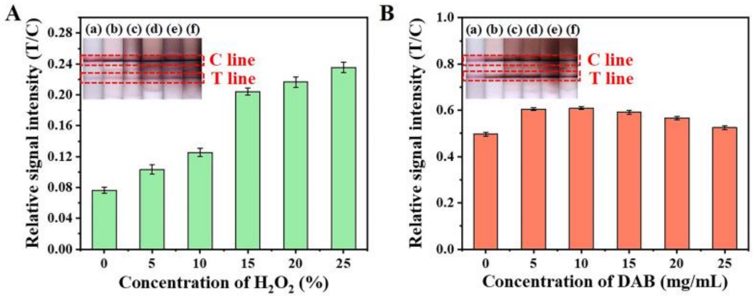

Figure S2. (A) Optimization of H2O2 concentration. Inset: Photographs of Apt-LFAs with different H2O2 concentrations. (a), (b), (c), (d), (e), and (f) are 0%, 5%, 10%, 15%, 20%, and 25%, respectively (n=3). (B) Optimization of DAB concentration. Inset: Photographs of Apt-LFAs with different DAB concentrations. (a), (b), (c), (d), (e), and (f) are 0 mg/mL, 5 mg/mL, 10 mg/mL, 15 mg/mL, 20 mg/mL, and 25 mg/mL, respectively (n=3).

Table S1. Nucleic acid sequence for kanamycin Apt-LFA

DNA name	Sequence (5'-3')
Aptamer	Biotin-TGGGGGTTGAGGCTAAGCCGATTTTT
PolyA-DNA	AAAAAAAAAAAAAAATTTTTAAAAAAATCGGCTTAGC
SH-DNA	SH-AGGGGCGGGGCGGGGCGGGGGC
DNAC	Biotin-AAAAATCACTCTATAATAATAAT

Underlined or bold italicized sequences indicate complementary sequences.

Table S2. Comparison of analytical properties of various kanamycin sensors

Detection method	Liner ranges	LOD	Signal amplification methods	Reference
Fluorescence	0.1–300 nmol/L	46.1 pmol/L	Coupling CHA with branched HCR	[4]
Fö rsterresonance energy transfer	50-500 nmol/L	28 nmol/L	-	[5]
Colorimetric	0.1–0.5 μmol/L	0.6 μmol/L	-	[6]
Colorimetric	1-100 nmol/L	1.49 nM	-	[7]
Lateral flow strip biosensor	1-30 nmol/L	0.0778 nmol/L	Silver nanoparticles as a signal amplification element	[8]
Lateral flow strip biosensor	5-500 nmol/L	4.96 nmol/L	Magnetic nanoparticles based separation and enrichment	[9]
Lateral flow strip biosensor	25-800 nmol/L	14.8 nmol/L	CRISPR/Cas12a	[10]
Lateral flow strip biosensor	1-250 ng/mL (1.72-429.1nM)	0.08 ng/mL (0.137nM)	AuNPs@polyA-cDNA/ GpG-Cu^{2+}	Our work

1. Frens, G., Controlled nucleation for the regulation of the particle size in monodisperse gold suspensions. *Nature Phys Sci* **1973,** 241, (105), 20-22.
2. Grabar, K. C.; Freeman, R. G.; Hommer, M. B.; Natan, M. J., Preparation and characterization of Au colloid monolayers. *Anal Chem* **1995,** 67, (4), 735-743.
3. Zhang, X.; Liu, B.; Dave, N.; Servos, M. R.; Liu, J., Instantaneous attachment of an ultrahigh density of nonthiolated DNA to gold nanoparticles and its applications. *Langmuir* **2012,** 28, (49), 17053-17060.
4. Liao, L.; Li, X.; Jiang, B.; Zhou, W.; Yuan, R.; Xiang, Y., Cascaded and nonlinear DNA assembly amplification for sensitive and aptamer-based detection of kanamycin. *Analytica Chimica Acta* **2022,** 1204, 339730.
5. Umrao, S.; Anusha, S.; Jain, V.; Chakraborty, B.; Roy, R., Smartphone-based kanamycin sensing with ratiometric FRET. *Rsc Adv.* **2019,** 9, (11), 6143-6151.
6. Tang, Y.; Hu, Y.; Zhou, P.; Wang, C.; Tao, H.; Wu, Y., Colorimetric detection of kanamycin residue in foods based on the aptamer-enhanced peroxidase-mimicking activity of layered WS2 nanosheets. *Journal of Agricultural and Food Chemistry* **2021,** 69, (9), 2884-2893.
7. Sharma, T. K.; Ramanathan, R.; Weerathunge, P.; Mohammadtaheri, M.; Daima, H. K.; Shukla, R.; Bansal, V., Aptamer-mediated ‘turn-off/turn-on’nanozyme activity of gold nanoparticles for kanamycin detection. *Chemical communications* **2014,** 50, (100), 15856-15859.

8. Liu, J.; Zeng, J.; Tian, Y.; Zhou, N., An aptamer and functionalized nanoparticle-based strip biosensor for on-site detection of kanamycin in food samples. *Analyst* **2018,** 143, (1), 182-189.
9. Ou, Y.; Jin, X.; Liu, J.; Tian, Y.; Zhou, N., Visual detection of kanamycin with DNA-functionalized gold nanoparticles probe in aptamer-based strip biosensor. *Anal Biochem* **2019,** 587, 113432.
10. Li, X.; Chen, X.; Mao, M.; Peng, C.; Wang, Z., Accelerated CRISPR/Cas12a-based small molecule detection using bivalent aptamer. *Biosensors and Bioelectronics* **2022,** 217, 114725.
